# Ethylene Response of Plum ACC Synthase 1 (*ACS1*) Promoter is Mediated through the Binding Site of Abscisic Acid Insensitive 5 (ABI5)  

**DOI:** 10.3390/plants8050117

**Published:** 2019-05-02

**Authors:** Avi Sadka, Qiaoping Qin, Jianrong Feng, Macarena Farcuh, Lyudmila Shlizerman, Yunting Zhang, David Toubiana, Eduardo Blumwald

**Affiliations:** 1 Department of Fruit Tree Sciences, ARO, The Volcani Center, 7528809 Rishon LeZion, Israel; mila@volcani.agri.gov.il; 2 Department of Plant Sciences, University of California, Davis, CA 95616, USA; qinqp@zafu.edu.cn (Q.Q.); fjr_agr@shzu.edu.cn (J.F.); mfarcuh@ucdavis.edu (M.F.); ytizhang@ucdavis.edu (Y.Z.); dtoubiana@ucdavis.edu (D.T.); eblumwald@ucdavis.edu (E.B.); 3 College of Horticulture, Sichuan Agricultural University, Chengdu 611130, China; 4 School of Ecological Technology and Engineering, Shanghai Institute of Technology, Shanghai 201418, China; 5 Xinjiang Production and Construction Corps Key Laboratory of Special Fruits and Vegetables Cultivation Physiology and Germplasm Resources Utilization, College of Agriculture, Shihezi University, Shihezi 832000, China

**Keywords:** transcription factor, TCP2, GL2, promoter

## Abstract

The enzyme 1-amino-cyclopropane-1-carboxylic acid synthase (ACS) participates in the ethylene biosynthesis pathways and it is tightly regulated transcriptionally and post-translationally. Notwithstanding its major role in climacteric fruit ripening, the transcriptional regulation of ACS during ripening is not fully understood. We studied fruit ripening in two Japanese plum cultivars, the climacteric Santa Rosa (SR) and its non-climacteric bud sport mutant, Sweet Miriam (SM). As the two cultivars show considerable difference in *ACS* expression, they provide a good system for the study of the transcriptional regulation of the gene. To investigate the differential transcriptional regulation of *ACS1* genes in the SR and SM, their promoter regions, which showed only minor sequence differences, were isolated and used to identify the binding of transcription factors interacting with specific *ACS1* cis-acting elements. Three transcription factors (TFs), abscisic acid-insensitive 5 (ABI5), GLABRA 2 (GL2), and TCP2, showed specific binding to the *ACS1* promoter. Synthetic DNA fragments containing multiple cis-acting elements of these TFs fused to β-glucuronidase (GUS), showed the ABI5 binding site mediated ethylene and abscisic acid (ABA) responses of the promoter. While TCP2 and GL2 showed constant and similar expression levels in SM and SR fruit during ripening, ABI5 expression in SM fruits was lower than in SR fruits during advanced fruit ripening states. Overall, the work demonstrates the complex transcriptional regulation of *ACS1*.

## 1. Introduction

Climacteric fruit ripening is characterized by a burst in respiration rates and increased levels of autocatalytic ethylene production [1,2]. In non-climacteric fruits, respiration declines gradually and there is no increase in ethylene production, although ethylene might play a role in various aspects of fruit maturation. Ethylene biosynthesis starts by the conversion of methionine into S-adenosyl-L-methionine (S-AdoMet), catalyzed by S-AdoMet synthase, followed by the synthesis of 1-amino-cyclopropane-1-carboxylic acid (ACC) by ACC synthase (*ACS*) and its conversion into ethylene by ACC oxidase (reviewed in [3,4]). The reaction catalyzed by *ACS* is a rate-limiting step in ethylene biosynthesis, therefore *ACS* plays an important role in different aspects of plant development and the responses to environmental cues. Transcriptional regulation of *ACS* genes can be mediated by transcription factors like Ripening Inhibitor (RIN) in tomato [5,6,7,8], C2H2 zinc finger proteins (C2H2-ZFPs) in banana [9], or Ethylene Response Factors (ERFs) in apple [10]. *ACS* activity can be also regulated post-translationally (reviewed in [3,11,12]); protein stability is affected by ubiquitination and phosphorylation/dephosphorylation by mitogen activated protein (MKP)-kinases [13]. Epigenetic regulation might also play a role in *ACS* expression [14].

*ACS* is encoded by a multigene family; for instance, eight members in *Arabidopsis* and tomato, four in cucumber, and three in pineapple [15,16,17,18]. Members are differentially expressed during plant development and in response to different cues [18]. In *Arabidopsis*, *AtACS6* is up-regulated by abscisic acid (ABA), brassinolide (BL), and indoleacetic acid (IAA), while *AtACS10* is down-regulated by ABA, IAA, methyl jasmonate (MeJA), and gibberellins (GA) [19]. In tomato, four gene members are induced during fruit ripening [15]. In members of the *Rosaceae* family, the importance of *ACS1* in fruit ripening was demonstrated. Apple *MdACS1* is predominantly expressed during fruit ripening and is associated with ethylene production [20,21,22], and levels of ethylene production were associated with different allelic forms of apple *ACS1* [20,21,23,24]. For instance, late *MdACS1-2/2* genotypes produced low ethylene, and the fruit could be stored longer than other genotypes [23]. Induction in peach *Pp-ACS1* mRNA levels was associated with ethylene production during fruit ripening. However, in stony-hard peach cultivars, characterized by low ethylene production, *ACS1* transcript levels were reduced [25,26]. 

Japanese plum (*Prunus salicina* L.) cultivars can display different ripening behaviors; climacteric, non-climacteric, and suppressed-climacteric [27,28]. Comparative analyses of climacteric Early Golden Japanese Plum and suppressed climacteric Shiro cultivars showed differential expression of four *ACS* genes that correlated with the fruit responses to ethylene [27]. Moreover, fruit ripening was in agreement with the expression of genes associated with ethylene perception (*Ethylene Receptor, ETR, ETR-related gene, ERS, Constitutive Triple Response, CTR*) and signaling (ERFs) [29]. Sweet Miriam (SM), a bud sport mutant of the climacteric plum cultivar Santa Rosa (SR), displayed a non-climacteric fruit ripening behavior [28,30,31,32,33,34]. As a result, SM showed a longer pit hardening stage (S2), and required 40 d extra to reach the mature stage (S3) and an additional 80 d and 100 d to achieve the commercial harvest stage (S4-I) and the fully ripe stage (S4-II), respectively [30]. SM fruit maintained significantly lower and constant ethylene production rates during ripening on-the-tree and during post-harvest storage, while SR fruit displayed a significant increase in ethylene production from mature stage S3/S4 until full ripening [34]. The increase in ethylene production during the climacteric SR fruit ripening was well correlated with the increased expression of *ACS1* and *ACS3,* while in the non-climacteric SM fruit, transcripts of both genes were a few orders of magnitude lower than in SR and remained constant throughout fruit ripening and post-harvest storage [34]. The copy number of two *ACS* genes was higher in SR than in SM, which may result in a higher expression of these genes in the climacteric cultivar [35].

The reduced expression of *ACS1* in SM, as compared to SR, might result from changes in the promoter region and/or alterations in the transcription factor(s) regulating gene expression, and might contribute to the differences between climacteric and non-climacteric fruits. Changes in the 1000 bp region, upstream to the transcription start site of *ACS1,* could not explain the differential *ACS1* expression between SR and SM. This region was used to identify transcription factors (TFs) interacting with *ACS1* cis-acting elements, and abscisic acid-insensitive 5 (ABI5), GLABRA 2 (GL2), and Teosinte Branched 1, Cycloidea, Pcf (TCP2) were isolated and their expression patterns analyzed. Although the TFs-expression patterns could not explain the differential *ACS1* expression in the two cultivars, our results suggest a complex regulation of *ACS1* expression by several TFs, some not shown previously to be involved in fruit ripening.

## 2. Materials and Methods

### 2.1. Plant Material

Leaves and fruits of Japanese plums (*Prunus salicina* L.) Santa Rosa (SR) and Sweet Miriam (SM) were collected, as previously described [28]. At total of four developmental stages, S2 (pit hardening), S3/S4 (mature); S4-I (commercial harvest); and S4-II (fully ripe) and 3 biological replicates with 6 fruits each, were used. All samples were frozen in liquid nitrogen immediately following collection and stored at −80 °C.

### 2.2. Isolation of ACS1 Promoter and Bioinformatics Analysis

Genomic DNA was extracted from SR and SM leaves using a CTAB-based method [36]. Degenerate primers, based on *ASC1* genes from peach (*Prunus persica*) (ppa004774 m.g) and from Japanese apricot (*Prunus mume*) (Pm018222ACS1) (http://www.phytozome.net/peach.php), were designed as follows: Forward primer, 5′-CAAGGACCAATAAGAGTGACAATTTCC(A/C/G/T)-3′, and the reverse primer, 5′-TTGCAATCTTGGAGAGTAA(A/C/G/T)AACC(A/C/G/T)-3′. The amplified products were cloned into a pCR-Blunt II-TOPO cloning vector (Invitrogen). Putative cis-acting elements and TFs binding to them were predicted using PlantCARE (http://bioinformatics.psb.ugent.be/webtools/plantcare/html/), PLACE (http://www.dna.affrc.go.jp/PLACE/), PlantPan 2.0 (http://plantpan2.itps.ncku.edu.tw/) and TSSPlant (http://www.softberry.com).

### 2.3. Promoter Activity Assays

The isolated *PsACS1* promoter *pPsACS1*, or tandem repeats of cis-acting elements (see below), were fused upstream of the *uidA* gene encoding β-glucuronidase (GUS) in a promoter-capture binary vector, *pCAMBIA1391Z*, and resulted in *pCAMBIA*-*pPsACS1-GUS*. The construct was introduced into *Agrobacterium* EHA105, followed by a transient transformation of fully expanded tobacco leaves, according to [37]. The *pCAMBIA1391Z* and *pCAMBIA1304* (*p35S-GUS*) were used as positive and negative controls, respectively. The infiltrated leaves were collected for analysis following 48 h incubation in the greenhouse. The effects of various compounds on promoter activity were tested by infiltration of the leaves with ethephon (10, 100 ppm), salicylic acid (SA) (1, 5 mM), and methyl jasmonate (MeJA) (10, 100 μM), 24 h after the *Agrobacterium*-infiltration. Treatments were performed at 4 ℃ for 36 h after *Agrobacterium*-infiltration. Each treatment was analyzed in three replicates (plants), each containing at least three individual leaves. Histochemical and fluorometric determination of GUS activity were performed, according to Zhang et al., 2009 [38]. Leaf proteins were extracted in an extraction buffer (50 mM NaPO_4_ buffer, pH 7.0, 10 mM DTT, 10 mM EDTA, 0.1% Sarcosyl, and 0.1% Triton X-100), and quantified using a Bradford reagent, according to the manufacturer’s instructions (BioRad). 

### 2.4. Screening with Yeast One-Hybrid Assay

Yeast one-hybrid assays were performed using Matchmaker^R^ (Takara Bio, Mountain View, CA, USA), according to thr manufacturer’s instructions. A single copy of *pPsACS1* was inserted upstream of the Aureobasidin A (*AbAr*) reporter gene in the *pAbAi* vector, resulting in the bait construct, *pAbAi*-*pPsACS1*. The forward primer used for bait vector construction was 5′-ACTGGGTACCCAAGGACCAATAAGAGTGAC-3′, and the reverse primer was 5′-CAGTCTCGAGCATCCATATCCACGTGTACACCAGTA-3′, both containing restriction enzyme sequences *KpnI* and *XhoI*. The *pAbAi*-*pPsACS1* was integrated into the genome of Y1HGold yeast strain and generated the bait-specific reporter strain, Y1HGold-*pAbAi*-*pPsACS*. To obtain the basal expression of bait reporter before library screening, the minimal inhibitory concentration of *AbAr* was determined in the absence of pray. The RNA of SR and SM fruits, isolated at S2 and S4 stages of development, was extracted, as described previously [34]. First-strand cDNAs were synthesized using the Matchmaker Gold Yeast -one-hybrid Library Screening System (Clontech). Four prey libraries, SR-S2, SR-S4, SM-S2, and SM-S4, were generated in yeast by co-transforming the purified ds cDNA and linear *pGADT-Rec* vector into the reporter yeast strain Y1HGold-*pAbAi*-*pPsACS1*. Positive colonies, obtained from library screenings, were further confirmed by re-streaking in selected media with *AbAr*, followed by the yeast colony PCR, using Matchmaker Insert Check PCR (Clontech). The PCR products were purified and sequenced using the T7 primer. The obtained sequences were annotated by BLASTX. Positive interaction candidate prey plasmids for reporter activation were rescued by isolating yeast plasmids and transformed into *E. coli*.

### 2.5. Verification of Positive Interactions using Yeast One-Hybrid Assay

Following the screening of the yeast libraries, three positive candidate TF clones were chosen for further analyses. Tandem repeats of cis-elements core sequences and corresponding mutant bait sequences were synthesized and sub-cloned into the reporter *pAbAi* vector (Table S1) using *KpnI* and *XhoI* restriction sites. The specific bait/prey and mutant/prey one-by-one interactions were performed after determining the minimal inhibitory concentration of *AbAr* for the bait strains.

### 2.6. Real-Time RT-PCR and Phylogenic Analysis of Transcription Factors

Expression analyses of the identified TFs were performed by quantitative PCR using an Applied Biosystems 7300 Real Time PCR System with three RNA replicates, each extracted from 20 fruits of three different trees, using a standard curve [28]. Primers were designed by Gene Script (Table S2). A gene encoding SAND protein-related trafficking protein (MON) was used as an internal reference [28,31]. For phylogenic analysis of the identified TFs, plant homologous sequences were retrieved from NCBI and phylogenetic analysis was performed by the maximum likelihood method using MEGA5 [39]. 

### 2.7. Statistical Analyses

Statistical analyses were carried out using JMP Pro 14.0.0 software (SAS Institute INC.) 

## 3. Results

### 3.1. Isolation and Prediction of ACS1 Promoter Regulatory Elements

The transcript of the Japanese plum *ACS1*, homologues to the peach prup.2g176900 (protein ppa004774 m.g), was the most abundant transcript of all *ACS* genes in SR fruit (Toubiana et al., unpublished). A 1144-bp fragment, upstream of the *PsACS1* gene translation initiation site (ATG), was isolated using primers designed and based on the peach *ACS1* gene (Figure S1). Alignment with the peach gene sequence (prup.2g176900) showed that this sequence included a 5′ untranslated region (5′UTR) of 294 bp and shared 93.5% identity with the peach *ACS1* corresponding sequence (not shown). This putative *PsACS1* promoter sequence was cloned and designated as *pPsACS1*. Analysis by PlantPAN showed that the promoter comprised several cis-acting regulatory elements, including those associated with the response(s) to ABA, ethylene, and gibberellins, as well as light responsiveness (Table S3). Since *ACS1* and *ACS3* transcripts in Sweet Miriam fruits, the non-climacteric mutant of SR, were remarkably lower than those in SR, and this reduction correlated well with their low ethylene contents [34], we investigated whether differences in the *ACS1* promoter region could explain the reduced *ACS1* expression. Using plum primers, the corresponding fragment was isolated from SM and showed almost 100% identity with the SR sequence, with only 2 bases, at positions −522 and −585, missing from the SM genome (Figure S1). Although we cannot exclude the possibility that differences between the SM- and SR-*ACS1* promoter sequence occur at a more upstream region, it seemed that the reduced *ACS1* expression in SM fruit was not a consequence of changes in its promoter sequence. 

### 3.2. pPsACS1 Basal Activity

**Figure 1:**
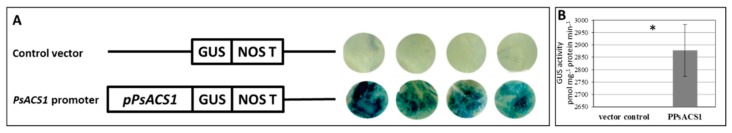
Transient expression of the native *1-amino-cyclopropane-1-carboxylic acid (ACC) synthase 1 (ASC1)* promoter, _P_PsACS1, in tobacco leaves. The constructs, *pPsACS1-β-glucuronidase* (*GUS)*, and a control vector were *Agrobacterium*-infiltrated into tobacco leaves (A) and analyzed for GUS activity for 48 h following transformation. Qualitative GUS activities of four replicates from each construct are shown (**A**). Their average mean values ± SE are shown in (**B**). (*) indicates significant difference in student t-test (*p* ≤ 0.01).

The activity of the *pPsACS1*-*GUS* fusion construct was assayed using transient expression in tobacco leaves (Figure 1). Following *Agrobacterium*-infiltration, the promoter conferred GUS activity, while no activity was detected by the empty vector. 

### 3.3. Isolation of Transcription Factors Interacting with pPsACS1

Isolation of putative TFs was carried out using the Yeast -one-hybrid screen. *pPsACS1* was cloned upstream of a reporter vector and introduced into the yeast genomic DNA, followed by the Yeast -one- hybrid screen using four fruit expression prey cDNA libraries. These libraries were constructed from SR and SM fruit at two developmental stages; pit hardening stage (SR-S2, SM-S2) and fully mature fruit (SR-S4 and SM-S4). Out of 4.8 × 10^6^–8.6 × 10^6^ screened clones from each library, 151, 196, 160, and 165 clones for SR-S2, SR-S4, SM-S2, and SM-S4, respectively, showed putative positive interactions following re-streaking (Table 1). All these clones were sequenced and annotated. About 25–30% of the clones encoded unknown proteins, while about 10% of the clones of each library encoded DNA- or RNA-binding proteins, without an apparent TF activity. The number of TF clones varied between the libraries, with SR-S2 showing the highest proportion of TFs (22%) and SM-S4 displaying the lowest proportion (about 5%). Overall, 69 clones encoding putative TFs were identified by the analysis in all four libraries. However, since some clones were present in more than one copy and some clones were common to more than a library, the overall number of unique TFs identified was 27 (Table S4). Next, we searched for putative binding sites of these 27 putative TFs in the *ACS1* promoter fragment using PlantPAN (plantpan.itps.ncku.edu.tw). A total of six TFs were identified as putatively having corresponding cis-acting elements in the promoter region: (1) XM_020567847.1 homologous to the peach protein ppa0198233m.g, a putative abscisic acid-insensitive 5, (2) XM_007202246.2 and XM_020568507.1, both showing homology to the peach protein ppa008024m.g, a putative G-Box-Binding Factor, (3) XM_007209140.2, showing homology to the peach protein ppa006484m.g., a putative bZIP transcription factor, (4) XM_007209393.2, homologous to the peach protein ppa009757m.g, a putative TF bHLH79, (5) XM_007211775.2 homologous to the peach protein ppa004612, a putative basic helix-loop-helix DNA-binding protein, TCP2, and (6) XM_007217098.2, homologous to the peach protein ppa001840m.g, a putative homeobox-leucine zipper protein, GLABRA 2. Notably, the GLABRA 2-like TF was present only in the library SR-S2 in seven copies. However, the other TFs were present in one or two copies in one or two of the libraries (Table S4). 

**Figure 2:**
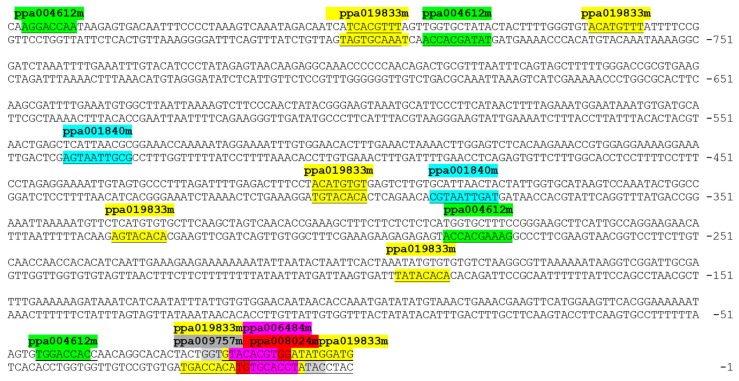
Putative binding sites of six transcription factors identified by the Yeast one-hybrid screen and predicted by PlantPAN in the promoter region of *ACS1* gene. The various transcription factors (TFs) are indicated by their *Prunus persica* protein ID above their putative binding sites. Binding sites of the same transcription factor are marked with same color.

The cis-acting elements of the six TFs are shown in Figure 2. Three of the TFs showed homology to the peach proteins ppa006484m.g, ppa008024m.g, and ppa009757m.g and displayed single binding sites immediately upstream of *ACS1* gene transcription start site, while the other three TFs, homologous to the peach proteins ppa0198233m.g (PsAB15), ppa004612m.g (PsTCP2), and ppa001840m.g (PsGL2) displayed multiple binding sites. Since multiple binding sites in the proximity to the transcription start site are considered to be more active [40], PsAB15, PsTCP2, and PsGL2 were selected for further analyses.

### 3.4. Interaction between PsAB15, PsTCP2, and PsGL2 and their Corresponding Cis-Acting Elements 

**Figure 3:**
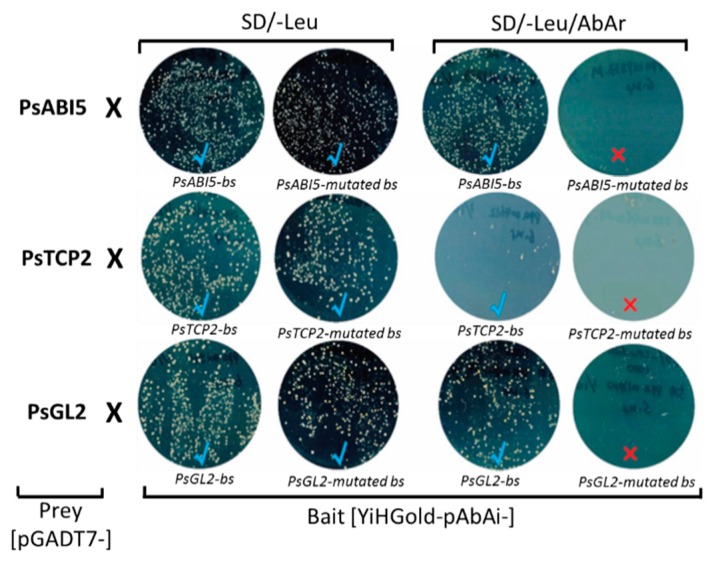
Confirmation of positive interactions by Yeast one-hybrid assay. Tandem repeats of cis-elements core sequences and their corresponding mutated sequences (Table S1), defined by the TFs bound to them, PsABI5, PsTCP2, and PsGL2, were synthesized and sub-cloned into reporter pAbAi vector (bait construct). The preys were prepared by extracting yeast plasmid from colonies showing positive interaction in the Yeast one-hybrid screen. A selective agar plate, SD/-Leu/AbAr (with AbAr above the minimal inhibitory concentration), was used to identify genuine positive interactions. The plates with distinct colonies are marked by “√” (blue), while the plates without distinct colonies are marked by “×” (red).

The interaction between PsAB15, PsTCP2, and PsGL2 and the *ACS1* promoter was validated using synthetic fragments containing biding sites in Yeast one-hybrid analyses. Design of the fragments was based on the putative binding sites for the given TF as predicted by PlantPAN. For instance, for PsGL2 (ppa001840m.g), PlantPAN predicted four binding sites (Figure 2). These four binding sites, including core sequences and two-three adjacent nucleotides, were synthesized in tandem. This sequence was repeated six times and was defined as *PsGL2-binding site* (*bs*) (Table S1). Similarly, fragments containing binding sites within the *ACS1* promoter of PsAB15 (3X, defined as *PsAB15-bs*) and PsTCP2 (4X, defined *PsTCP2-bs*) were also synthesized in tandem (Table S1). Mutations were carried on in core nucleotides of each binding site (defined as *PsGL2-mutated bs, PsTCP2-mutated bs, and PsGL2-mutated bs)*. These synthetic fragments and their corresponding mutated fragments were used as baits in a Yeast one-hybrid assay, using their corresponding TFs clones as preys (Figure 3). Bait strain containing tandem repeats of the non-mutated cis-element, activated the *AbAr* reporter on selective media SD/-Leu/AbA through co-transformation with the corresponding candidate prey strain. The co-transformation of the bait strains, containing *mutated*-*bs* of *pPsAB15* and *pPsTCP2* and the candidate prey strains, did not result in distinct colonies on SD/-Leu/AbA (Figure 3). For *pPsGL2-bs*, although small colonies were detected, the interaction was considered positive, compared to the number and size of the colonies obtained with the non-mutated cis-elements. 

### 3.5. Relative Expression of the TFs during Japanese Plum Fruit Development

**Figure 4:**
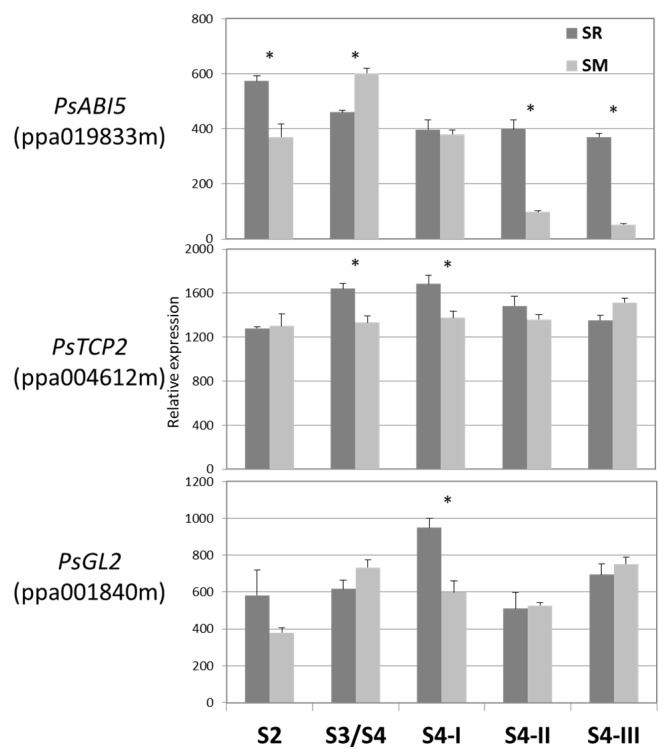
Expression levels of the indicated TFs (in parentheses, their *Prunus persica* protein ID) in climacteric Japanese plum Santa Rosa (SR) and non-climacteric Sweet Miriam (SM) during the different fruit developmental stages: S2 (pit hardening); S3/S4 (the second exponential growth phase), S4-I (commercial ripening), S4-II (fully ripen), and S4-III (overripe). Values are the mean ± SE (n = 3). (*) indicates significant difference between SM and SR in student t-test (*p* ≤ 0.05).

It has been shown that *ACS1* expression increased significantly during the transition from S3/S4 stage to S4-III in SR fruit, but not in SM fruit [33]. Transcript levels of *PsABI5* were slightly reduced in SR from the S2 to S4-III stages, while the expression was significantly lower in SM fruit at the S4-II and S4-III stages (Figure 4). Expression of *PsGL2* and *PsTCP2* was relatively unchanged during fruit development in both SR and SM cultivars. 

### 3.6. Basal in vivo Activity of pPsAB15, pPsTCP2, and pPsGL2 and their Corresponding Mutated Forms 

**Figure 5:**
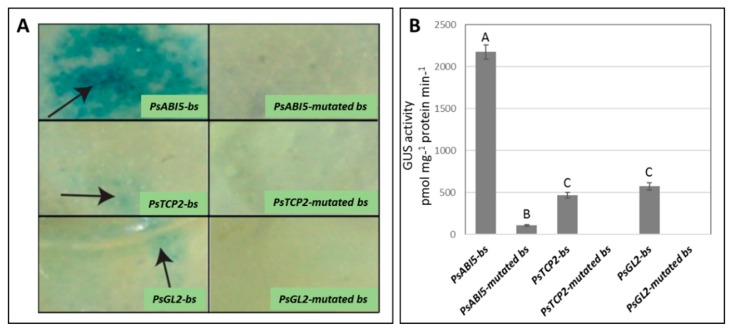
GUS activities generated by synthetic fragments containing tandem repeats of core promoter cis-elements and their corresponding mutated sequences (Table S1). The cis-acting elements are defined by the transcription factors bound to them, *PsABI5-bs* (binding site), *PsTCP2-bs,* and *PsGL2-bs* or their corresponding mutated forms, *PsABi5-mutated bs*, *PsTCP2-mutated bs,* and *PsGL2*-*mutated bs*. Qualitative (A) and quantitative (B) GUS activities, 48 h following *Agrobacterium* infiltration. Values are the mean ± SE (n = 3). Different letters indicate significant difference in Tukey–Kramer test (*p* ≤ 0.05).

The three synthetic promoter fragments and their corresponding mutated forms were cloned in front of the GUS reporter gene and introduced into *Agrobacterium*. *Agrobacterium*-infiltration was carried on into tobacco leaves and GUS activity was assessed (Figure 5). The GUS basal activity driven by *PsAB15-bs* was three-four-fold higher than the activity conferred by *PsTCP2*-bs and *PsGL2-bs*. The transformations with the corresponding mutated forms of all three synthetic promoters resulted in no significant GUS activity (Figure 5). 

### 3.7. Effect of Hormone and other Treatments on GUS Activities Driven by pACS1, Synthetic Promoter Fragments, and their Mutated Forms

**Figure 6:**
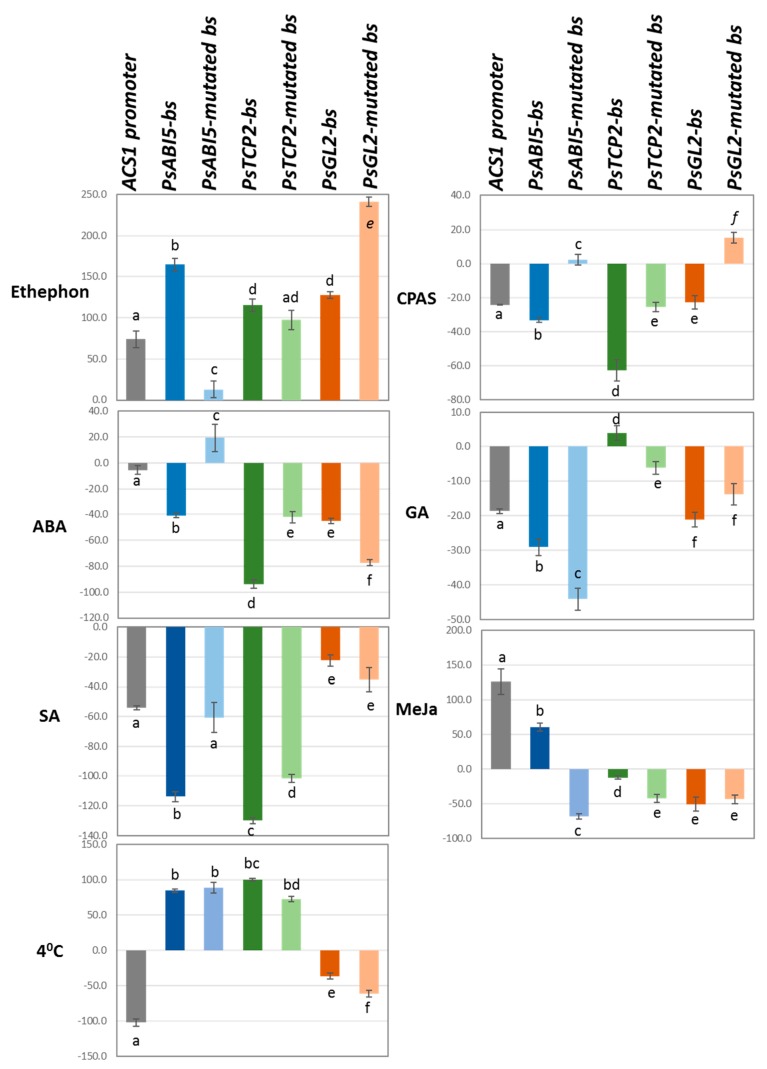
Effects of several treatments on GUS activity conferred by the *ACS1* promoter and synthetic fragments containing tandem repeats of cis-elements and their mutated forms. Synthetic fragments were defined according to the TF binding to them (binding site (bs)). Net GUS activities (pmol mg^−1^ protein min^−1^) are shown following subtraction of activities generated by mock-treated leaves and by the empty vector. Activities were measured in *Agrobacterium*-infiltrated tobacco leaves following spraying with the indicated compounds: ethephon, 3-cyclopropyl-1-enyl-propanoic acid salt (CPAS), abscisic acid (ABA), gibberellic acid (GA), methyl jasmonate (MeJa), salicylic acid (SA), or following cold treatment (4 °C). Values are the mean ± SE (n = 3). Different letters indicate significant differences in the Tukey–Kramer test (*p* ≤ 0.05).

The GUS activity of *Agrobacterium*-infiltrated plants with the *ACS1* native promoter region or synthetic promoters containing *PsAB15 bs, PsTCP2-bs,* and *PsGL2*-*bs,* and their mutated forms, was assessed following hormone, chemical, and cold treatments (Figure 6). Notably, net activities were lower than those presented in Figure 1 and Figure 5, because they were performed following short incubations of 30–45 min, as opposed to 48 h (Figure 1 and Figure 5). As expected, ethephon (releasing ethylene) resulted in increased GUS activity driven by the native *ACS1* promoter, *pACS1*. An additional 2.5-fold increased activity was detected by the synthetic fragment containing the *PsABI5*-*bs,* and a remarkable decreased activity was seen by its mutated form. These results suggested that this element was involved in the response to ethylene. The synthetic fragments containing *PsTCP2-bs* and *PsGL2*-*bs* also induced GUS activity; however, their mutated forms also resulted in similar activities, indicating that the action of *PsTCP2-bs* and *PsGL2*-*bs* was not biologically relevant (Figure 6). Treatment with 3-cyclopropyl-1-enyl-propanoic acid salt (CPAS), a water-soluble inhibitor of ethylene signaling [41], reduced the GUS activity driven by the *pACS1*, with a slight additional reduction by the synthetic fragment containing the *PsABI5-bs*. The mutated form of this cis-element resulted in a slight increase in GUS activity, suggesting that the CPAS action was mediated by *PsABI5*. Activity of the *PsTCP2*-*bs* and its mutated form generated a similar reduction in activity, indicating its biological irrelevance. The *PsGL2-bs* also reduced GUS activity, similarly to the *ACS1* native promoter and its mutated form resulted in the induction of GUS activity, suggesting the involvement of this cis-element in mediating CPAS action. ABA treatments also resulted in reduced *pACS1*-driven GUS activity, with an 8-fold further decrease conferred by the *PsABI5-bs* and the induction of activity by its mutated form, suggesting the possible involvement of this element in the response to ABA. MeJa treatments induced *pACS1*-driven GUS activity. A synthetic promoter containing *PsABI5*-*bs* also generated induced activity, although this effect was 2.5-fold lower than that generated by the full-size promoter. The mutated form resulted in reduced GUS activity, suggesting the possible involvement of *PsABI5* in the MeJa effect. GA, SA, and cold (4 °C) treatments reduced *pACS1*-driven GUS activity, however the similar effects generated by the synthetic fragments and their mutated forms questioned their biological relevance. 

## 4. Discussion

The transcriptional regulation of *ACS* genes is one of the regulation processes required to tightly control ethylene production rates. Regardless of its importance as a major enzyme controlling ethylene biosynthesis, transcriptional gene regulation, especially during fruit ripening, is complex. Members of a gene family can respond differently to distinct cues, probably through specific cis- and trans-acting factors. Although plum fruit provides a particular case, isolation of plum TFs affecting *ACS1* transcription and identification of how their transcript levels vary between SR and SM, might contribute to the understanding of ACS1 regulation during fruit ripening. 

### 4.1. ACS1 Promoter Activity in Response to Various Treatments

The combined use of the bioinformatics analysis of *ACS1* gene upstream sequences and the non-biased Yeast one-hybrid screen led to the identification of three transcription factors, *PsAB15*, *PsTCP2,* and *PsGL2*, and their corresponding cis-acting elements. Alignments of their amino acid sequences with the corresponding sequences from other plant species showed high similarities (Figure S2). The high similarity levels allowed studying the activity of the *ACS1* promoter region and synthetic fragments containing cis-acting elements of *PsAB15*, *PsTCP2,* and *PsGL2* in tobacco leaves using an *Agrobacterium*-mediated infiltration. A ~850 bp fragment, upstream to the transcription start site of the gene, showed remarkable activity. It can be speculated that the removal of leaves and the preparation of leaf-disks was sufficient to induce activity, probably due to wounding response [3,42]. Ethephon treatments induced GUS activity, while CPAS, a soluble ethylene inhibitor, suppressed it. Similarly, ethylene treatments during fruit post-harvest storage increased *ACS1* transcripts, while 1-methylcyclopropane (1-MCP ) treatments reduced *ACS1* expression [34]. Thus, the overall response of *ACS1* from plum was similar to that of other studied plant species, including *Arabidopsis AtACS4* and *AtACS7* [43,44]. Ethephon and MeJa treatments resulted in induced *ACS1*-driven GUS activity, as seen on tomato fruit and sugarcane with co-induction of *ACS* expression [45,46]. On the other hand, ABA, GA, SA, and cold, suppressed *ACS1* promoter activity. It has been shown that ABA induced climacteric and non-climacteric fruit ripening by promoting ethylene biosynthesis (reviewed in [47,48]). The exogenous application of ABA resulted in induced *ACS* expression in *Arabidopsis* seedlings and during the ripening of tomato, strawberry, and mango fruit [38,43,49,50]. However, our results showing that ABA treatments reduced *ACS* expression are similar to those reported in kiwifruit [51]. We recently showed that ABA levels were remarkably induced in SM during ripening on-the-tree, as reported for other non-climacteric plum cultivars [52], while no such induction was observed for SR fruits [34].

### 4.2. Activity of Synthetic Promoter Fragments and their Mutated Forms

The use of synthetic tandem repeats and their mutated forms allowed the characterization of the physical interaction of the TFs with their corresponding cis-acting elements. Induced GUS activity was only considered when the TF generated a similar or higher activity than the full promoter and no GUS activity was induced by the mutated form of the TF. The induced expression by ethephon, and the repression by the ethylene inhibitor CPAS, appeared to be mediated by the ABI5 binding element. Interestingly, both ABA and MeJa generated promoter responses through this cis-acting element.

### 4.3. Interactions among ABI5, Ethylene, and ABA 

ABA-insensitive, ABI5, belongs to a group of basic leucine zipper class of transcriptional regulators, containing conserved leucine residues within the zipper region near the C terminus (Figure S2). Among other ABI genes, *ABI1-4*, was originally identified as an *Arabidopsis* mutant, which was insensitive to ABA inhibition of seed germination (reviewed in [53,54]). *ABI3-5* are TFs mediating ABA response in many species, while *ABI1-2* are type 2C phosphatases. ABI5 is associated with the regulation of developmental processes, seed germination, lateral root growth, leaf senescence, and the responses to environmental cues [53,55], mediating the crosstalk with other hormones during abiotic stress. At the transcriptional level, ABI5 is regulated by WRKY18, WRKY40, WRKY60, MYB7, and other TFs, including ABI3 and ABI4 [53,56]. At the posttranslational level, it undergoes modifications, including phosphorylation/dephosphorylating and ubiquitination [53]. Our results, showing that an ABI5-like protein binds to the *ACS1* promoter, suggests that it could also play a role in fruit ripening. The ethylene response factor, ERF 96, was shown to be associated with the response of *Arabidopsis* to ABA and the regulation of ABI5 expression, supporting a possible connection between ABI5 and ethylene homeostasis [57]. ABI5 transcripts were remarkably reduced in SM fruits during the advanced ripening stages, S4-II and S4-III. This reduction might play a role in ripening arrest during SM fruit development [34]. The activation of the *ACS1* promoter activity by ethylene, ABA, and possibly by MeJa appeared to be mediated through the ABI5-binding site. 

### 4.4. Binding of GL2 and TCP2 to the ACS1 Promoter

The *ACS1* promoter also interacted with TCP2 and GL2, although these TFs did not appear to mediate a response to any of the treatments and showed constant and similar transcript levels during the development of SM and SR fruits. TCP2 is part of a large family of TFs, associated with many aspects of plant growth and development, including cell proliferation, shoot apical meristem, leaf development, biosynthesis of phytohormones, etc. (reviewed in [58,59]). They interact with many TFs, including ethylene response factors (ERFs). The expression of some TCPs correlated well with the ripening of peach and banana fruit [60,61]. Moreover, virus-induced gene silencing of TCP2A in banana resulted in induced ethylene levels associated with enhanced *ACS1* transcription [61]. GL2 contains three conserved helixes present in DNA-binding factors (Figure S1). GL2 was shown to be associated with suppressing root hair development, trichome development, and pattern formation in leaves [62,63]. Although there are no indications showing that GL2 regulates *ACS1* expression, GL2 participates in fruit ripening. GL2 also interacts, albeit indirectly, with ethylene, suppressing the expression of Root Hair Defective 6 (RHD6) [62], which reacts with ethylene insensitive 3 (EIN3) to promote root hair development. 

## 5. Conclusions

We have shown previously that ethylene biosynthesis, as well as its autocatalytic production, were remarkably induced during the climacteric SR plum fruit ripening and the increased expression of genes associated with ethylene biosynthesis, while the opposite occurred in the non-climacteric mutant, SM cultivar [28,30,31,33,34]. Here we demonstrated the possible involvement of three TFs, ABI5, GL2, and TCP2, in *ACS1* transcription. Although our findings could not be directly associated with the differences in ethylene production in SM and SR, they suggest that the regulation of *ACS1* expression is a complex mechanism involving several TFs. Two of these TFs, GL2 and ABI5, were not previously shown to be involved in fruit ripening. 

## Figures and Tables

**Table 1:** Summary of clones isolated by the Yeast one-hybrid screen using the pPsACS1 bait strain.

		Expression library							
		SR-S2		SR-S4		SM-S2		SM-S4	
		Number of Clones	%	Number of Clones	%	Number of Clones	%	Number of Clones	%
Total screened clones		4,800,000		8,600,000		4,800,000		6,800,000	
Positive clones		151		196		160		165	
Annotation of positive clones	Unknown	41	27.2%	46	23.5%	39	24.4%	53	32.1%
	Known proteins of no apparent relevance	64	42.4%	117	59.7%	92	57.5%	86	52.1%
	Transcription factors	33	21.9%	17	8.7%	11	6.9%	8	4.8%
	DNA/RNA binding	13	8.6%	16	8.2%	18	11.3%	16	9.7%

## Supplementary Materials

The following are available online at https://www.mdpi.com/2223-7747/8/5/117/s1: Figure S1: Alignment of 5′-UTR and −842 bp upstream region of Santa Rosa (SR) and Sweet Miriam (SM) *ACS1* gene. Transcription start site is marked by +1. First ATG is at position +295. Figure S2: Amino acid sequence alignment and phylogenetic analyses of ABI5, TCP2, and GL2. The conserved domains are aligned by Clustal. The leucine residues in the zipper region are boxed for ABI5. Conserved helixes are labeled for TCP2 and GL2. Species names are followed by their NCBI ID. The evolutionary history is inferred by using the Maximum Likelihood method, based on the JTT matrix-based model. Initial tree(s) for the heuristic search are obtained automatically as the following: When the number of common sites is <100 than one fourth of the total number of sites, the maximum parsimony method is used; otherwise BIONJ method with MCL distance matrix is used. The tree is drawn to scale, with branch lengths measured in the number of substitutions per site. The analysis for ABI5 involved 9 amino acid sequences, 11 sequences for TCP2, and 10 sequences for GL2. All positions containing gaps and missing data are eliminated. A total number of 384 positions in the final dataset are for ABI5, 379 positions for TCP2, and 725 positions for GL2. Evolutionary analyses are conducted in MEGA5.
