# Supplementary material for: Ethylene Response of Plum ACC Synthase 1 (ACS1) Promoter is Mediated through the Binding Site of Abscisic Acid Insensitive 5 (ABI5)
Source: Plants (Basel). 2019 May 2;8(5):117. doi: 10.3390/plants8050117 (PMC6572237; doi:10.3390/plants8050117)
Supplement: Supplementary file 1 [file plants-08-00117-s001.pdf]

Figure S1

Fig. S1. Alignment of 5'-UTR and -842 bp upstream region of Santa Rosa (SR) and Sweet Miriam (SM) *ACS1* gene. Transcription start site is marked by +1. First ATG is at position +295.

```
SR      ATAAGAGTGACAATTTCCCCTAAAGTCAAATAGACAATCATCACGTTTAGTTGGTGCTAT -782
SM      ATAAGAGTGACAATTTCCCCTAAAGTCAAATAGACAATCATCACGTTTAGTTGGTGCTAT
        ************************************************************

SR      ACTACTTTTGGGTGTACATGTTTATTTTCCGGATCTAAATTTTGAAATTTGTACATCCCT -722
SM      ACTACTTTTGGGTGTACATGTTTATTTTCCGGATCTAAATTTTGAAATTTGTACATCCCT
        ************************************************************

SR      ATAGAGTAACAAGAGGCAAACCCCCCAACAGACTGCGTTTAATTTCAGTAGCTTTTTGGG -662
SM      ATAGAGTAACAAGAGGCAAACCCCCCAACAGACTGCGTTTAATTTCAGTAGCTTTTTGGG
        ************************************************************

SR      ACCGCGTGAAGAAGCGATTTTGAAATGTGGCTTAATTAAAAGTCTTCCCAACTATACGGG -602
SM      ACCGCGTGAAGAAGCGATTTTGAAATGTGGCTTAATTAAAAGTCTTCCCAACTATACGGG
        ************************************************************

SR      AAGTAAATGCATTCCCTTCATAACTTTTAGAAATGGAATAAATGTGATGCAAACTGAGCT -542
SM      AAGTAAATGCATTCCC-TCATAACTTTTAGAAATGGAATAAATGTGATGCAAACTGAGCT
        **************** *******************************************

SR      CATTAACGCGGAAACCAAAAATAGGAAAATTTGTGGAACACTTTGAAACTAAAACTTGGA -482
SM      CATTAACGCGGAAACCAAA-ATAGGAAAATTTGTGGAACACTTTGAAACTAAAACTTGGA
        ******************* ****************************************

SR      GTCTCACAAGAAACCGTGGAGGAAAAGGAAACCTAGAGGAAAATTGTAGTGCCCTTTAGA -422
SM      GTCTCACAAGAAACCGTGGAGGAAAAGGAAACCTAGAGGAAAATTGTAGTGCCCTTTAGA
        ************************************************************

SR      TTTTGAGACTTTCCTACATGTGTGAGTCTTGTGCATTAACTACTATTGGTGCATAAGTCC -362
SM      TTTTGAGACTTTCCTACATGTGTGAGTCTTGTGCATTAACTACTATTGGTGCATAAGTCC
        ************************************************************

SR      AAATACTGGCCAAATTAAAAATGTTCTCATGTGTGCTTCAAGCTAGTCAACACCGAAAGC -302
SM      AAATACTGGCCAAATTAAAAATGTTCTCATGTGTGCTTCAAGCTAGTCAACACCGAAAGC
        ************************************************************

SR      TTTCTTCTCTCTCATGGTGCTTTCCGGGAAGCTTCATTGCCAGGAAGAACACAACCAACC -242
SM      TTTCTTCTCTCTCATGGTGCTTTCCGGGAAGCTTCATTGCCAGGAAGAACACAACCAACC
        ************************************************************

SR      ACACATCAATTGAAAGAAGAAAAAAAAATATTAATACTAATTCACTAAATATGTGTGTGTC -182
SM      ACACATCAATTGAAAGAAGAAAAAAAAATATTAATACTAATTCACTAAATATGTGTGTGTC
        ************************************************************

SR      TAAGGCGTTAAAAAATAAGGTCGGATTGCGATTTGAAAAAAGATAAATCATCAATATTTA -122
SM      TAAGGCGTTAAAAAATAAGGTCGGATTGCGATTTGAAAAAAGATAAATCATCAATATTTA
        ************************************************************

SR      TTGTGTGGAACAATAACACCAAATGATATATGTAAACTGAAACGAAGTTCATGGAAGTTC -62
SM      TTGTGTGGAACAATAACACCAAATGATATATGTAAACTGAAACGAAGTTCATGGAAGTTC
        ************************************************************

SR      ACGGAAAAAAATAGTGTGGACCACCAACAGGCACACTACTGGTGTACACGTGGATATGGAT -2
SM      ACGGAAAAAAATAGTGTGGACCACCAACAGGCACACTACTGGTGTACACGTGGATATGGAT
        ************************************************************
         +1
SR      GGATATCAACGTACAACACGCAAGCAATTACGCCCATTGACCAATATTAACACAATTAAA +59
SM      GGATATCAACGTACAACACGCAAGCAATTACGCCCATTGACCAATATTAACACAATTAAA
        ************************************************************

SR      TTCTGTTATTTCTATTCTGGGAGGTCGGTTTTTTCCAAAGTTGAACTTTGAAAAGAAAAA +119
SM      TTCTGTTATTTCTATTCTGGGAGGTCGGTTTTTTCCAAAGTTGAACTTTGAAAAGAAAAA
        ************************************************************

SR      AAAAACCTTTGAAAACTTTTGCTTCCCCTCCTGCTTGCCAATCTGCCATCGCCTATAAAT +179
SM      AAAAACCTTTGAAAACTTTTGCTTCCCCTCCTGCTTGCCAATCTGCCATCGCCTATAAAT
        ************************************************************

SR      TCCCACTACTATCTCACCCATTGCCCCCACAATTGATAGCTTGCTTGCTTGCAAACCTCA +239
SM      TCCCACTACTATCTCACCCATTGCCCCCACAATTGATAGCTTGCTTGCTTGCAAACCTCA
        ************************************************************

SR      CTAAAAATTCCACTAATATTATTCTTGTGTGTGAATATTCTTCGGAACCAAGAAAATG   +297
SM      CTAAAAATTCCACTAATATTATTCTTGTGTGTGAATATTCTTCGGAACCAAGAAAATG
        **********************************************************
```

Fig S2. Amino acid sequence alignment and phylogenetic analyses of ABI5, TCP2 and GL2. The conserved domains were aligned by Clustal. The leucine residues in the zipper region are boxed for ABI5. Conserved helixes are labeled for TCP2 and GL2 (Martín-Trillo and Cubas., 2010; Rerie et al., 1994). Species names are followed by their NCBI ID. The evolutionary history was inferred by using the Maximum Likelihood method based on the JTT matrix-based model. Initial tree(s) for the heuristic search are obtained automatically as the following. When the number of common sites is < 100 than one fourth of the total number of sites, the maximum parsimony method is used; otherwise BIONJ method with MCL distance matrix is used. The tree is drawn to scale, with branch lengths measured in the number of substitutions per site. The analysis for ABI5 involved 9 amino acid sequences, 11 sequences for TCP2, 10 sequences for GL2. All positions containing gaps and missing data are eliminated. A total number of 384 positions in the final dataset are for ABI5, 379 positions for TCP2, and 725 positions for GL2. Evolutionary analyses is conducted in MEGA5 (Tamura et al., 2011).

Figure S2

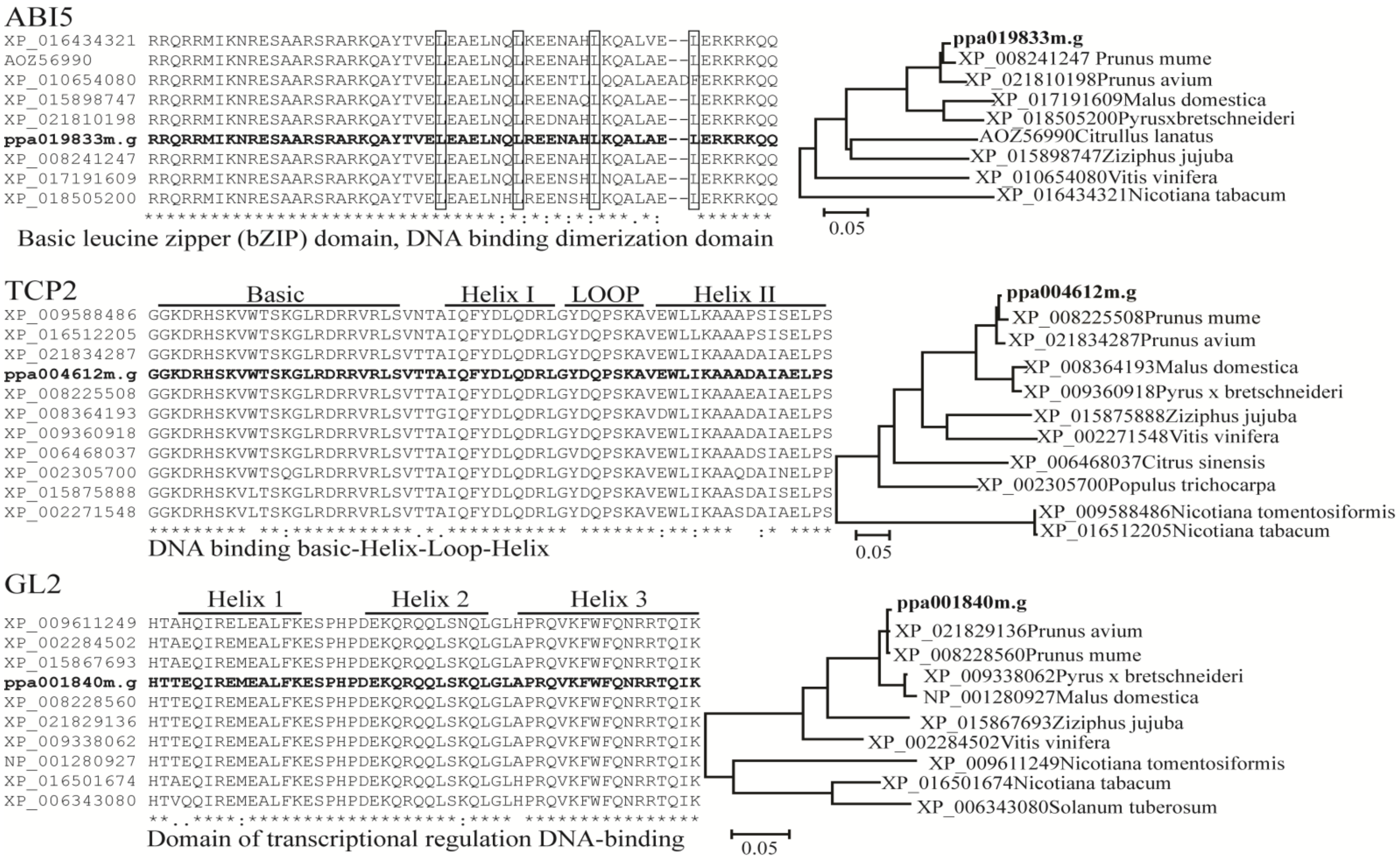

ABI5
XP_016434321 RRQRRMIKNRESAARSRARKQAYTVELEAELNQLKEENAHLKQALVE--IERKRKQQ
AOZ56990 RRQRRMIKNRESAARSRARKQAYTVELEAELNQLREENAHLKQALAE--IERKRKQQ
XP_010654080 RRQRRMIKNRESAARSRARKQAYTVELEAELNQLKEENTLLQQALAEADFERKRKQQ
XP_015898747 RRQRRMIKNRESAARSRARKQAYTVELEAELNQLREENAQLKQALAE--IERKRKQQ
XP_021810198 RRQRRMIKNRESAARSRARKQAYTVELEAELNQLREDNAHLKQALAE--IERKRKQQ
ppa019833m.g RRQRRMIKNRESAARSRARKQAYTVELEAELNQLREENAHLKQALAE--IERKRKQQ
XP_008241247 RRQRRMIKNRESAARSRARKQAYTVELEAELNQLREENAHLKQALAE--IERKRKQQ
XP_017191609 RRQRRMIKNRESAARSRARKQAYTVELEAELNQLREENSHLNQALAE--IERKRKQQ
XP_018505200 RRQRRMIKNRESAARSRARKQAYTVELEAELNHLREENSHLKQALAE--IERKRKQQ
Basic leucine zipper (bZIP) domain, DNA binding dimerization domain
ppa019833m.g
XP_008241247 Prunus mume
XP_021810198Prunus avium
XP_017191609Malus domestica
XP_018505200Pyrusxbretschneideri
AOZ56990Citrullus lanatus
XP_015898747Ziziphus jujuba
XP_010654080Vitis vinifera
XP_016434321Nicotiana tabacum
0.05
TCP2
Basic
Helix I
LOOP
Helix II
XP_009588486 GGKDRHSKVWTSKGLRDRRVRLSVNTAIQFYDLQDRLGYDQPSKAVEWLLKAAAPSISELPS
XP_016512205 GGKDRHSKVWTSKGLRDRRVRLSVNTAIQFYDLQDRLGYDQPSKAVEWLLKAAAPSISELPS
XP_021834287 GGKDRHSKVWTSKGLRDRRVRLSVTTAIQFYDLQDRLGYDQPSKAVEWLIKAAADAIAELPS
ppa004612m.g GGKDRHSKVWTSKGLRDRRVRLSVTTAIQFYDLQDRLGYDQPSKAVEWLIKAAADAIAELPS
XP_008225508 GGKDRHSKVWTSKGLRDRRVRLSVTTAIQFYDLQDRLGYDQPSKAVEWLIKAAAEAIAELPS
XP_008364193 GGKDRHSKVWTSKGLRDRRVRLSVTTGIQFYDLQDRLGYDQPSKAVDWLIKAAADAIAELPS
XP_009360918 GGKDRHSKVWTSKGLRDRRVRLSVTTAIQFYDLQDRLGYDQPSKAVEWLIKAAADAIAELPS
XP_006468037 GGKDRHSKVWTSKGLRDRRVRLSVTTAIQFYDLQDRLGVDQPSKAVEWLIKAAADSIAELPS
XP_002305700 GGKDRHSKVWTSQGLRDRRVRLSVTTAIQFYDLQDRLGYDQPSKAVEWLIKAAQDAINELPP
XP_015875888 GGKDRHSKVLTSKGLRDRRVRLSVTTAIQFYDLQDRLGYDQPSKAVEWLIKAASDAISELPS
XP_002271548 GGKDRHSKVLTSKGLRDRRVRLSVTTAIQFYDLQDRLGYDQPSKAVEWLIKAASDAIAELPS
DNA binding basic-Helix-Loop-Helix
ppa004612m.g
XP_008225508Prunus mume
XP_021834287Prunus avium
XP_008364193Malus domestica
XP_009360918Pyrus x bretschneideri
XP_015875888Ziziphus jujuba
XP_002271548Vitis vinifera
XP_006468037Citrus sinensis
XP_002305700Populus trichocarpa
XP_009588486Nicotiana tomentosiformis
XP_016512205Nicotiana tabacum
0.05
GL2
Helix 1
Helix 2
Helix 3
XP_009611249 HTAHQIRELEALFKESPHPDEKQRQQLSNQLGLHPRQVKFWFQNRRTQIK
XP_002284502 HTAEQIREMEALFKESPHPDEKQRQQLSKQLGLAPRQVKFWFQNRRTQIK
XP_015867693 HTAEQIREMEALFKESPHPDEKQRQQLSKQLGLAPRQVKFWFQNRRTQIK
ppa001840m.g HTTEQIREMEALFKESPHPDEKQRQQLSKQLGLAPRQVKFWFQNRRTQIK
XP_008228560 HTTEQIREMEALFKESPHPDEKQRQQLSKQLGLAPRQVKFWFQNRRTQIK
XP_021829136 HTTEQIREMEALFKESPHPDEKQRQQLSKQLGLAPRQVKFWFQNRRTQIK
XP_009338062 HTTEQIREMEALFKESPHPDEKQRQQLSKQLGLAPRQVKFWFQNRRTQIK
NP_001280927 HTTEQIREMEALFKESPHPDEKQRQQLSKQLGLAPRQVKFWFQNRRTQIK
XP_016501674 HTAEQIREMEALFKESPHPDEKQRQQLSKQLGLHPRQVKFWFQNRRTQIK
XP_006343080 HTVQQIREMEALFKESPHPDEKQRQQLSKQLGLHPRQVKFWFQNRRTQIK
Domain of transcriptional regulation DNA-binding
ppa001840m.g
XP_021829136Prunus avium
XP_008228560Prunus mume
XP_009338062Pyrus x bretschneideri
NP_001280927Malus domestica
XP_015867693Ziziphus jujuba
XP_002284502Vitis vinifera
XP_009611249Nicotiana tomentosiformis
XP_016501674Nicotiana tabacum
XP_006343080Solanum tuberosum
0.05

Table S1. Synthetic promoters containing sequences of *cis*-element core sequences and corresponding mutated sequences for one-by-one bait/prey interactions

Target *Prunus persica* gene IDs	Name of bait constructions	Synthesized sequence (5'-3')	# of tandem repeats
ppa019833m	*PsABI5-binding site (bs)*	ATCACGTTTAGTACATGTTTATCTACATGTGTGATCTCATGTGTGCAAATATGTGTGTGTGTACACGTGGATACACGTGGATATGTACACGTGGATACACGTGGAT	3×
	PsABI5-mutated-binding site (bs)	ATCTGATTTAGTAGTTGTTTATCTAGTTGACTGATCTCATGACTGCAAATATGACTGTGACTACTGCTGGATACTACTGGATATGTACTGCTGGATACTGCTGGAT	Mutant ,3×
ppa004612m	*PsTCP2-binding site (bs)*	CAAGGACCAATAGTTGGTGCTATACTCATGGTGCTTTCCGTGTGGACCACCA	4×
	PsTCP2-mutated binding site (bs)	CAAGCTGCAATAGTTGCACCTATACTCATGCACCTTTCCGTGTGCTGCACCA	Mutant ,4×
ppa001840m	*PsGL2-binding site (bs)*	GCTCATTAACGCGGGTGCATTAACTACT	6×
	PsGL2-mutated binding site (bs)	GCTCAAATACGCGGGTGCAAATACTACT	Mutant ,6×

Table S2. Primers used for qPCR analyses

Primer Name	Sequence (5'-3')	*Prunus persica* gene IDs
PsABI5-F	AGCCAGGAAACAGGCATACACA	ppa019833m.g
PsABI5-R	TAGCTTTCTGGGCCCTGCTC	ppa019833m.g
PsTCP2-F	CGGTCCGAGATTCGGGTGAA	ppa004612m.g
PsTCP2-R	GCTGGTGGTGGTGAGCAATG	ppa004612m.g
PsGL2-F	ACAGGCACACCACTGAGCAA	ppa001840m.g
PsGL2-R	GGCGCTCTTGTATGGCCTTG	ppa001840m.g
MON-F	GGGAACCTTATATTGGCGTAGG	
MON-R	CTGCTGACGAGGACTACTTATTG	

Table S3. *cis*-elements in P_{PsACS1} as predicted by PlantCARE

***cis*-elements**	**Sequences**	**Position/Strand**	**Function**
5'UTR Py-rich stretch	TTTCTTCTCT	549/+,621/-	cis-acting element conferring high transcription levels
AAGAA-motif	GAAAGAA	620/+	/
ABRE	CACGTG	834/-	cis-acting element involved in the abscisic acid responsiveness
ACE	ACGTGGA	835/+	cis-acting element involved in light responsiveness
ARE	TGGTTT	320/-	cis-acting regulatory element essential for the anaerobic induction
Box 4	ATTAAT	637/-	light responsiveness
Box I	TTTCAAA	109,955,350, 976, 207,700/-	light responsive element
Box-W1	TTGACC	885/+	fungal elicitor responsive element
CAAT-box	CCAAT/CAAT/CAATT/CAAAT/	6,7,19,35,43,488,499,615,720,739,748,873,889,890,901,1006,1007,1058/+;115,337,410,411,473,584,616,617,693,699,728,884,1048,1059,1060/-	common cis-acting element in promoter and enhancer regions
CATT-motif	GCATTC	257/+	part of a light responsive element
ERE	ATTTCAAA	109,207/-	ethylene-responsive element
G-Box	CACGTT/TAAACGTG	50/+,834/-; 50/-	cis-acting regulatory element involved in light responsiveness
GARE-motif	TCTGTTG	154/-	gibberellin-responsive element
GT1-motif	GTGTGTGAA	1115/+	light responsive element
I-box	GATATGG	840/+	part of a light responsive element
LTR	CCGAAA	541/+	cis-acting element involved in low-temperature responsiveness
MNF1	GTGCCC(A/T)(A/T)	417/+	light responsive element
Sp1	CC(G/A)CCC	149/+,927/-	light responsive element
TATA-box	TATA/TTTTA/TAATA/ATATAT/TATAAAT/	66,128,241,273,639,1020,1022,1102,1105/+;225,358,504,636,677,754,755,893,1090/-	core promoter element around -30 of transcription start
TATCCAT/C-motif	TATCCAT	847/-	/
W box	TTGACC	885/+	/
chs-Unit 1 m1	ACCTACCACAC	602/+	part of a light responsive element

Table S4. List of transcription factors (TF) and their number (#) in the various libraries, SR S2, SR S4, SM S2 and SM S4, along with their *Prunus persica* protein ID, and annotations in *P. persica* and *Arabidopsis*. TFs in bold were predicated by PlantPan as having binding sites in the promoter of *ACS1*.

SR S2		SR S4		SM S2		SM S4		*Prunus persica* protein ID	*Prunus persica* annotation	*Arabidopsis* annotation
#	NCBI ID	#	NCBI ID	#	NCBI ID	#	NCBI ID			
1	XM_020567027.1	1	XM_020567027.1					ppa002612m.g	Prunus persica AP2-like ethylene-responsive transcription factor ANT	DRG [Arabidopsis thaliana]
1	XM_020567870.1							ppa001612m.g	Prunus persica transcriptional corepressor LEUNIG (LOC18770397), transcript	AP2/EREBP transcription factor [Arabidopsis thaliana]
		**1**	**XM_020567847.1**					**ppa019833m.g**	**Prunus persica protein ABSCISIC ACID-INSENSITIVE 5**	**Basic-leucine zipper (bZIP) transcription factor family protein [Arabidopsis**
		1	XM_007201172.2					ppa013219m.g	Prunus persica CLAVATA3/ESR (CLE)-related protein 41 (LOC18766746),	no hits
**1**	**XM_007202246.2**			**1**	**XM_020568507.1**			**ppa008024m.g**	**Prunus persica G-box-binding factor 1 (LOC18771192), transcript variant X1,**	**G-box binding factor 1 [Arabidopsis lyrata subsp. lyrata]**
1	XM_007202528.2							ppa011596m.g	Prunus persica high mobility group B protein 2 (LOC18770435), mRNA	NFD2 [Arabidopsis thaliana]
						1	XM_007205702.2	ppa010331m.g	Prunus persica 14-3-3-like protein GF14 kappa (LOC18772503), mRNA	general regulatory factor 8 [Arabidopsis thaliana]
		1	XM_007207945.2					ppa003727m.g	Prunus persica chromatin assembly factor 1 subunit FAS2 (LOC18773563), mRNA	Transducin/WD40 repeat-like superfamily protein [Arabidopsis thaliana]
		**1**	**XM_007209140.2**			**1**	**XM_007209140.2**	**ppa006484m.g**	**Prunus persica bZIP transcription factor 16 (LOC18775920), mRNA**	**hypothetical protein ARALYDRAFT_473396 [Arabidopsis**
						**2**	**XM_007209393.2**	**ppa009757m.g**	**Prunus persica transcription factor bHLH79 (LOC18776453), transcript**	**transcription factor BIG PETAL P (BPE) [Arabidopsis thaliana]**
				**1**	**XM_007211775.2**	**1**	**XM_007211775.2**	**ppa004612m.g**	**Prunus persica transcription factor TCP2 (LOC18779022), transcript**	**putative basic helix-loop-helix DNA binding protein TCP2, partial**
**7**	**XM_007217098.2**							**ppa001840m.g**	**Prunus persica homeobox-leucine zipper protein GLABRA 2**	**same**
2	XM_007218446.2	2	XM_007218446.2					ppa012424m.g	Prunus persica high mobility group B protein 1 (LOC18785344), mRNA	NFD2 [Arabidopsis thaliana]
1	XM_007225154.2							ppa001977m.g	Prunus persica protein CHROMATIN REMODELING 19 (LOC18792817),	SNF2 domain-containing protein / helicase domain-containing protein [Arabidopsis
1	XM_007225901.2							ppa012509m.g	Prunus persica 50S ribosomal protein L18, chloroplastic (LOC18791554), mRNA	Z-box binding factor 3 [Arabidopsis thaliana]

						1	XM_020554291.1	ppa001493m.g	Prunus persica calmodulin-binding transcription activator 5 (LOC18792253),	
		1	XM_020561047.1					ppa018846m.g	Prunus persica glycosyltransferase family 92 protein RCOM_0530710	zinc finger family protein [Arabidopsis lyrata subsp. lyrata]
		1	XM_020564500.1					ppa014801m.g	PREDICTED: Prunus persica zinc finger MYM-type protein 1-like	no hits
2	XM_020565363.1	4	XM_020565363.1	1	XM_020565363.1			no	Prunus persica ribonuclease P protein subunit p25-like protein (LOC18789816),	Alba DNA/RNA-binding protein [Arabidopsis thaliana]
2	XM_020565367.1			1	XM_020565367.1			no	Prunus persica ribonuclease P protein subunit p25-like protein (LOC18789816),	Alba DNA/RNA-binding protein [Arabidopsis thaliana]
						1	XM_020567936.1	ppa012912m.g	Prunus persica calmodulin-7 (LOC18770340), mRNA	Z-box binding factor 3 [Arabidopsis thaliana]
				1	XM_007209089.2			ppa005912m.g	Prunus persica AP-2 complex subunit mu (LOC18775895), mRNA	
				1	XM_020566022.1			ppa000612m.g	Prunus persica calmodulin-binding transcription activator 3 (LOC18773983),	Calmodulin-binding transcription activator 3 [Arabidopsis thaliana]
				1	XM_007207935.2			ppa014789m.g	Prunus persica scarecrow-like protein 30 (LOC18775211), mRNA	GRAS family transcription factor [Arabidopsis thaliana]
				1	XM_020566232.1			ppa002392m.g	Prunus persica scarecrow-like protein 14 (LOC18773542), mRNA	GRAS family transcription factor [Arabidopsis thaliana]
14	XR_002272096.1	4	XR_002272096.1	3	XR_002272096.1	1	XR_002272096.1	ppa011892m.g	Prunus persica uncharacterized LOC18790550 (LOC18790550),	signal transducer/transcription protein, putative (DUF1685) [Arabidopsis thaliana]
